# Net meta-analysis: comparison of bare metal stent, drug-coated balloon and drug-eluting stent in the treatment of cerebral arterial stenosis

**DOI:** 10.3389/fneur.2025.1637301

**Published:** 2026-01-05

**Authors:** Shaowen Xu, Xue Dong, Qizhi Zhang

**Affiliations:** The Fifth People’s Hospital of Jinan, Shandong Second Medical University, Jinan, China

**Keywords:** bare metal stent, drug-coated balloon, drug-eluting stent, intracranial artery stenosis, network meta-analysis, vertebral artery stenosis

## Abstract

**Objective:**

Previous studies compared drug-eluting stents (DES) or drug-coated balloons (DCB) with bare metal stents (BMS), but no direct comparisons of the three devices exist. This network meta-analysis assesses outcomes differences among DES, DCB, and BMS in cerebral arterial stenosis.

**Methods:**

We screened literature from PubMed, Embase, Web of Science, and Cochrane databases published from January 1, 2010 to March 9, 2025, on clinical studies comparing the three devices. Two researchers independently screened the articles using Endnote software, assessed their quality using the Newcastle-Ottawa Scale (NOS) and Cochrane Risk of Bias 2.0 tools, and performed STATA 14.0 with the “network” command.

**Results:**

Meta-analysis indicated associations in restenosis between DCB and BMS (odds ratio (OR): 0.24, 95%confidence interval (CI): 0.10–0.57), DES and BMS (OR: 0.37, 95%CI: 0.22–0.64). However, no significant difference was observed between DCB and DES. There was no statistical difference among different types of instruments in teach success. DCB was associated with higher risks of rescue procedures compared to BMS (OR: 3.41, 95%CI: 1.13–10.25). However, DCB was linked to reduction in perioperative complications compared to both BMS (OR: 0.3, 95%CI: 0.12–0.93) and DES (OR: 0.43, 95%CI: 0.19–0.95). In subgroup analysis by follow-up times, BMS was correlated with higher risks of restenosis compared with both DCB (OR: 9.06, 95% CI: 2.43–33.83) and DES (OR: 3.71, 95%CI: 1.27–10.83) within 6 months. Beyond 6 months, while DCB showed a favorable trend in restenosis compared to BMS, the advantage associated with DES was more pronounced (OR: 2.38, 95%CI: 1.19–4.75). BMS was linked to a higher risk of restenosis compared to DES in both intracranial artery stenosis (ICAS) (OR: 4.32, 95%CI: 2.45–7.62) and vertebral artery stenosis (VAS) (OR: 2.40, 95%CI: 1.25–4.59).

**Conclusion:**

In ICAS or VAS patients, DCB appeared to demonstrate comparable efficacy to DES in reducing restenosis, with both potentially superior to BMS. The advantage of DCB may be more pronounced within 6 months, whereas DES may exhibit potential sustained benefits beyond this period. DCB may also be associated with fewer perioperative complications, though they carried a higher likelihood of requiring urgent stent replacement. The comparable outcomes between DCB and DES suggested that both may be valuable endovascular options, forming a basis for clinical decision-making pending further evidence.

## 1 Introduction

Globally, intracranial artery stenosis (ICAS) is a major contributor to stroke morbidity, with a lower prevalence in Caucasians compared to Chinese populations (20.0% vs. 43.0%) (1). Additionally, up to 20% of vertebral artery stenosis cases result in posterior circulation strokes (2). The Stenting and Aggressive Medical Management for Preventing Recurrent Stroke (SAMMPRIS) Trial (3) and the Vitesse Intracranial Stent Study for Ischaemic Stroke Therapy (VISSIT) Trial (4) both demonstrated that aggressive drug therapy is superior to stenting in preventing recurrent strokes. Effect of Stenting Plus Medical Therapy vs. Medical Therapy Alone on Risk of Stroke and Death in Patients With Symptomatic Intracranial Stenosis (CASSISS) Trial (5) with optimized patient and operator selection, found that stenting plus medicine was non-inferior to medicine alone, but no superior. Balloon Angioplasty vs. Medical Management for Intracranial Artery Stenosis (BASIS) Trial (6) was a breakthrough, standalone balloon angioplasty (without stenting) was superior to medicine management alone in preventing stroke recurrence. However, some patients continue to experience recurrence or worsening of symptoms despite pharmacological treatment, making endovascular therapy a valuable complementary option.

The Stenting Registry Study and the Wingspan Stent System Post Market Surveillance (WEAVE) trial have reported perioperative complication rates ranging from 2.6 to 4.3%, suggesting that intracranial stenting may be safe in carefully selected patients with ICAS (7–10), representing a significant advancement in cerebrovascular therapy. Studies have shown that in-stent restenosis rates range from 20 to 30%, particularly in intracranial and vertebral arteries (11–13). This condition can hinder the restoration of blood flow and increase the risk of further ischemic events, such as strokes or transient ischemic attacks.

With continuous advancements in technology, two new treatment modalities DES and DCB have emerged. Both modalities feature a drug-eluting surface but differ in their mechanisms of drug release (14–16). DCB, in particular, offers the inherent advantage of being “implant-free” (17). In 2023, a meta-analysis by Wu et al. (18) suggested that angioplasty using DCB might be the most effective treatment for vertebral artery stenosis (VAS), a leading cause of stenosis in this region. A meta-analysis indicated that DES and BMS have comparable safety profiles for treating ICAS and VAS (19). However, no direct comparison of DES, DCB, and BMS has been performed. Therefore, we conducted a network meta-analysis to assess DCB as an alternative to DES.

## 2 Study methodology

This study adhered to the Preferred Reporting Items for Systematic Reviews and Meta-Analyses for Network Meta-Analyses (PRISMA-NMA) guidelines (20) and was prospectively registered in PROSPERO (CRD420250648228).

### 2.1 Literature search

We conducted a comprehensive literature search in PubMed, Embase, Web of Science, and Cochrane databases for clinical studies comparing the efficacy and safety of DCB, DES, and BMS in patients with cerebral artery stenosis. The search covered publications from January 1, 2010 to March 9, 2025. Key search terms included “drug-coated balloon,” “drug-eluting stent,” “bare metal stent,” “vertebral artery,” “intracranial,” and “stenosis.” In addition, relevant literature was manually reviewed and included.

### 2.2 Literature screening

The network-meta-analysis was conducted according to the PICO framework.

#### 2.2.1 Eligibility criteria

##### 2.2.1.1 Population (P)

Patients with ICAS or VAS confirmed by digital subtraction angiography (DSA). Studies enrolling patients with in-stent restenosis as the primary condition were excluded.

##### 2.2.1.2 Interventions (I) and comparators (C)

Studies with pairwise or multi-arm comparisons of DCB, DES, and BMS.

##### 2.2.1.3 Outcome (O)

Effectiveness: Restenosis (RS), including in-stent restenosis (ISR), as diagnosed by neurointerventionalists using DSA, computed tomography angiography (CTA), or Doppler ultrasound (DUS); Elective procedure: Stent implantation performed ≥14 days after the qualifying ischemic event. Safety: Technical success: the criteria used in each included study; Rescue procedure: unplanned intervention performed during or within 24 h after the index procedure because of acute complications (in-stent thrombosis, flow-limiting dissection, or sudden neurological deterioration). If the study states that all devices were successfully deployed without acute complications, the case is deemed rescue-free; perioperative complications, including death, stroke, transient ischemic attack, hemorrhagic stroke or acute thrombosis within 30 days. Follow-up durations: not less than 3 months.

##### 2.2.1.4 Study design

Randomized controlled trials (RCTs), cohort studies, or case-control studies were eligible for inclusion. Each group included a minimum of five subjects.

#### 2.2.2 Screening method

Endnote software was used and two researchers independently screened the records in duplicate. When conflicts arose, the two researchers negotiated and resolved them, and if they could not be resolved, a third researcher (QZ) adjudicated.

### 2.3 Data extraction

The following information was extracted from each study: first author’s name, year of publication, study region, study design, interventions and comparators, sample size, mean age, gender distribution, follow-up duration, comorbidities, restenosis rates, surgical success rates, and perioperative complications. For continuous data presented as median and range or median and interquartile range, values were converted to mean ± standard deviation using the methods proposed by Wan et al. (21) and Luo et al. (22). All scaffolds without drug coatings were categorized as BMS. Where available, we also recorded whether the stenting procedure was performed electively or as a rescue intervention. However, due to inconsistent reporting across studies, we were unable to stratify the analysis by this variable. Data extraction was performed independently by two investigators. Any discrepancies were resolved through consultation with a third investigator (QZ).

### 2.4 Quality evaluation

The quality of cohort studies was assessed using the Newcastle–Ottawa Scale (NOS) (23), with scores categorized as follows: 7–9 (high quality), 4–6 (moderate quality), and 0–3 (low quality). Randomized controlled trials (RCTs) were evaluated using the Cochrane Risk of Bias 2.0 (RoB 2.0) tool (24), which assesses the following domains: randomization process, deviations from intended interventions, missing outcome data, measurement of the outcome, selection of the reported result, and overall bias. Studies were classified as high risk of bias if one or more domains were rated as high risk. Given that this study evaluated interventional procedures, lack of blinding alone was not considered sufficient to classify a study as high risk of bias. Quality assessments were conducted independently by two reviewers, and disagreements were resolved through discussion.

### 2.5 Statistical analyses

**Figure 1:**
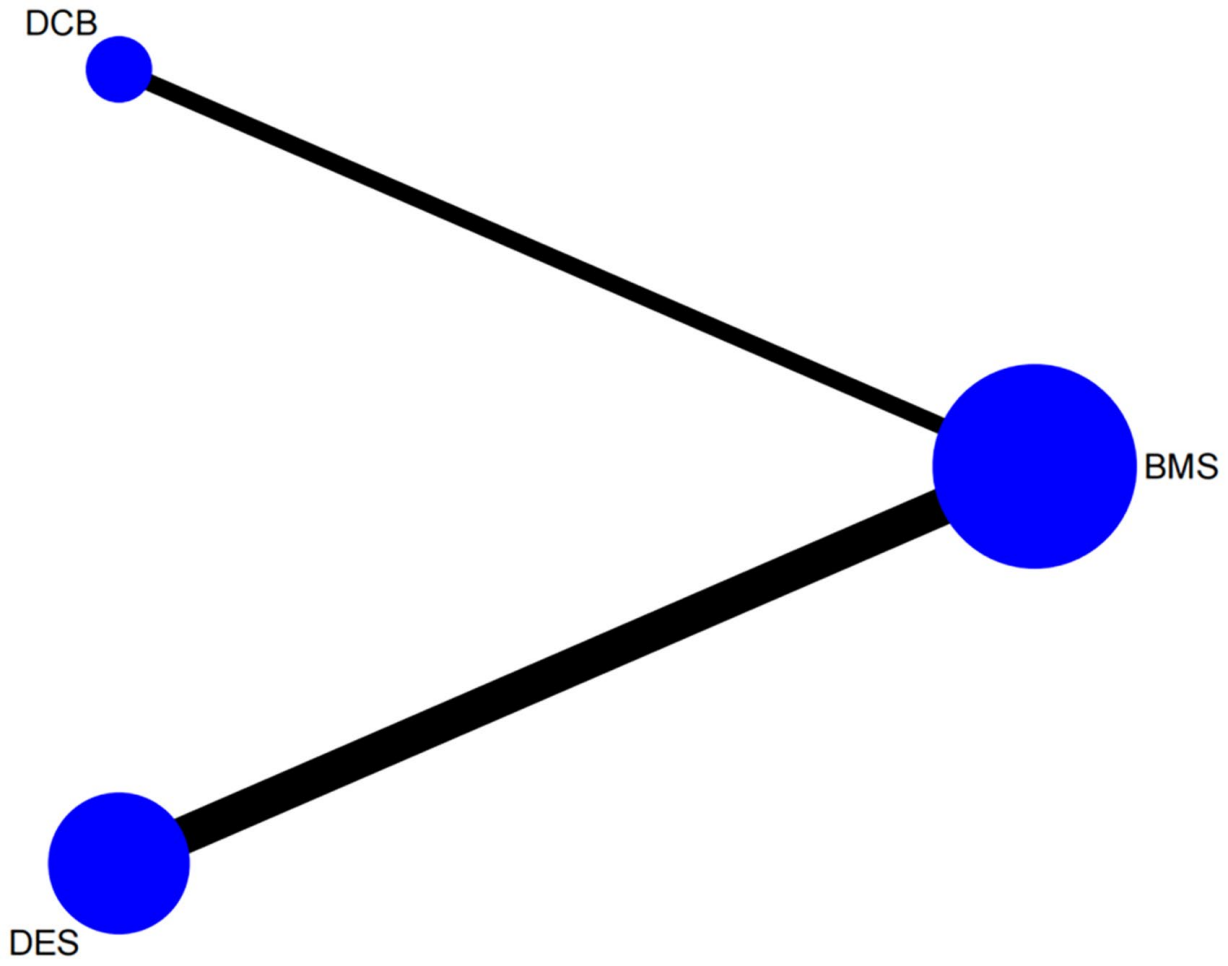
Network map.

All statistical analyses were performed using STATA 14.0 software. Continuous outcomes were pooled using weighted mean differences (WMD) with corresponding standard deviations (SD), while dichotomous outcomes were synthesized using odds ratios (OR), each reported with a 95% confidence interval (CI). A network plot was constructed using the network command when the number of studies in each group exceeded the number of nodes. When more than 10 studies were included per comparison group, small-study effects and potential publication bias were assessed through visual inspection of funnel plots. To compare the relative efficacy and safety of DCB, DES, and BMS, a league table was employed to present the indirect and direct comparison results. The network geometry in this study formed a simple star-shaped structure (Figure 1), with no direct comparisons between DCB and DES, precluding the possibility of conducting an inconsistency test. To explore potential sources of heterogeneity, we performed univariable meta-regression analyses for the DES versus BMS comparison, which included a sufficient number of studies (*n* = 11). We examined the following pre-specified study-level covariates: study region, lesion location, study design, and follow-up duration. We examined the following pre-specified study-level covariates: study region, lesion location, study design, and follow-up duration. We adhered to the methodological recommendation of including more than 10 studies to minimize the risk of overfitting, for meta-regression analyses targeting the DES vs. BMS comparison (*n* = 11) (25). Given the heterogeneity in the definition of restenosis across the included studies, we adopted the following strategy to enhance the robustness of our findings: we first performed the network meta-analysis on a dataset of 16 studies that explicitly reported specific criteria for restenosis. Subgroup analysis was conducted to evaluate the consistency of the results by restricting the analysis to a more homogeneous subset of 12 studies that uniformly applied the ≥50% stenosis. According to previous findings, DES has demonstrated superiority over BMS in treating in-stent restenosis in vertebral arteries (19). Based on these findings, we proposed the following *a priori* hypothesis: considering the differences in follow-up time points and stenosis sites, DES was still superior to BMS different follow-up times: up to 6 months (including the 6th month) and more than 6 months; different stenosis sites: VAS and ICAS. We used subgroup analysis for comparison.

## 3 Research findings

### 3.1 Results of literature search

**Figure 2:**
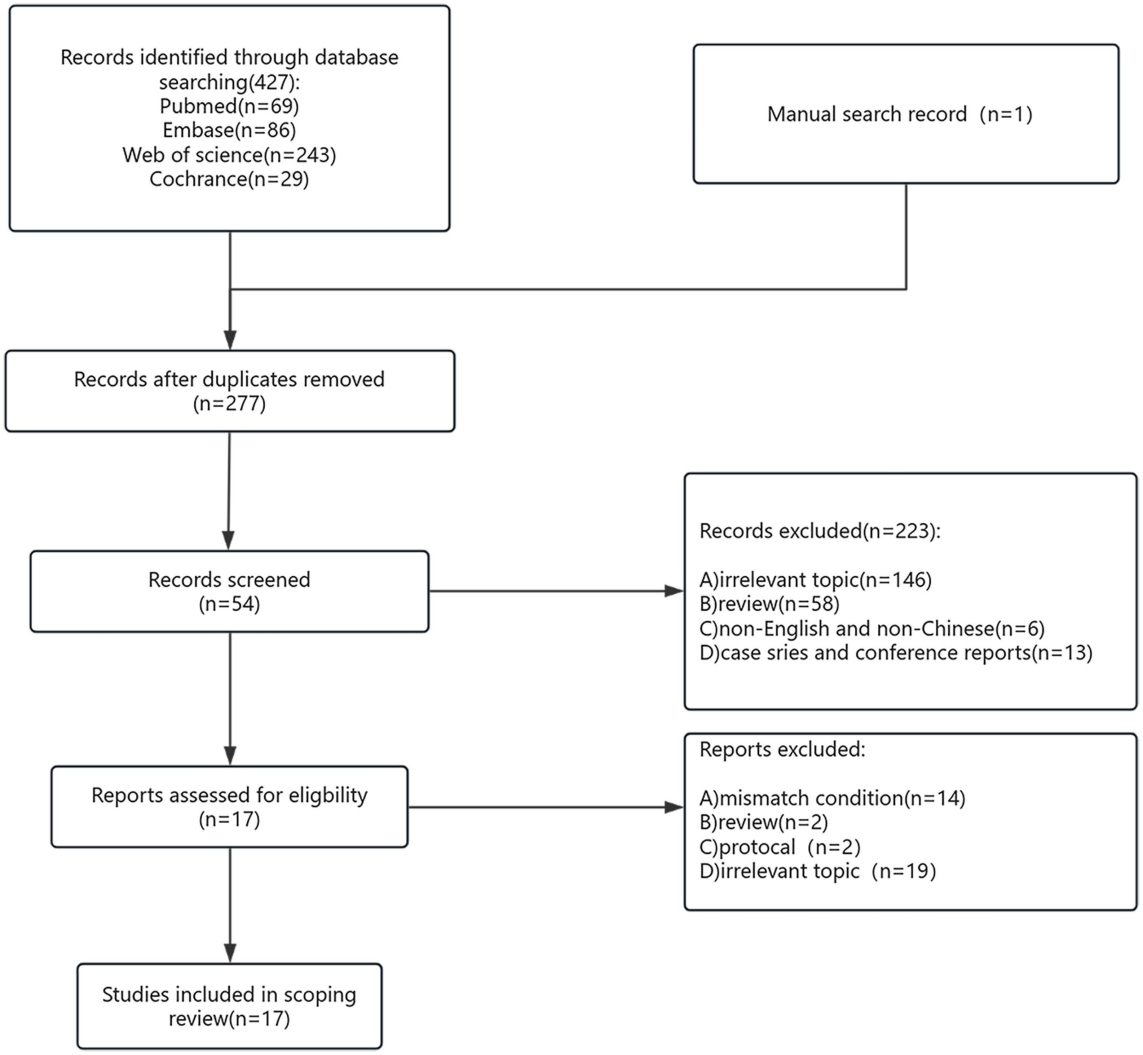
Flow chart of the system retrieval and selection process.

A total of 428 studies were initially identified through the systematic literature search. After the removal of duplicates, 277 studies remained for title and abstract screening. Of these, 54 full-text articles were assessed for eligibility. Ultimately, 17 studies met the inclusion criteria and were included in the final analysis (Figure 2), encompassing a total of 2,551 patients.

### 3.2 Basic characteristics of the included studies

Among the 17 studies included, 5 compared DCB versus BMS and 12 compared DES versus BMS. The majority of studies (76.5%, 13/17) were conducted in China, with a total of 2,511 patients included. A total of 2,580 patients were treated with balloon angioplasty and stenting (228 in the DCB group, 1,010 in the DES group, and 1,342 in the BMS group). Of the 2,580 subjects, 76.8% (1,982/2,580) were male, and 8.9% (203/2,580) had hypertension, as shown in Table 1. Among the studies, Feng et al. (26) compared DES, balloon-expanding stents, and self-expanding stents. In this study, all non-drug-eluting stents defined as BMS were included, and the data from these two groups were combined.

**Table 1:** Characteristics of the included studies.

Author	Year	Region	Study	Inclusion criteria		Sample, *n* DCB/DES BMS	
				Stenosis area	Intervention		
Philipp	2018	Switzerland	Retrospective cohort	ICA	DEB vs. BMS	8	11
Zhang	2020	China	Retrospective cohort	ICA	DCB vs. BMS	38	38
M-Y Wang	2021	China	RCT	VA	DCB vs. BMS	49	46
Bei Li	2024	China	Retrospective cohort	ICA	DCB vs. BMS	43	75
Ma	2025	China	RCT	ICA	DCB vs. BMS	90	90
Raghuram	2012	USA	Retrospective cohort	VA	DES vs. BMS	13	15
Song	2012	China	Prospective cohort	VA	DES vs. BMS	112	98
Nicolas	2013	Germany	Retrospective cohort	VA	DES vs. BMS	16	25
Feng	2018	China	Retrospective cohort	VA	DES vs. BMS	158	109

Author	Restenosis	Technical success	Perioperative complications	Imaging follow-up time, months	Duration of DAPT, months	Score
Philipp	>50%	Residual stenosis < 50%	Stroke or any death within 30 day	Mean 3 (DCB) Mean 4 (DES)	DCB:6 BMS:6	8
Zhang	>50% stenosis within or immediately adjacent (within 5 mm) of the treated segment and >20% absolute luminal loss	NG	Acute thrombosis, Any ischemic stroke and cerebral hemorrhage	Mean 6	DCB:3 BMS:6	9
M-Y Wang	≥50%	Completion of the procedure with <30% residual stenosis of the target lesion, without flow-limiting dissection and absence of adverse events within 1 month of the index procedure	NG	12	DCB:6 BMS:6	–
Bei Li	>50% stenosis of the luminal diameter in or within 5 mm of the treatment segment and absolute luminal loss >20%	Residual stenosis < 50%	Ischemic or hemorrhage stroke and death	Mean 12	DCB:3 BMS:3–6	9
Ma	(1) for the target lesion with postoperative residual stenosis <30%, follow-up angiography shows >50% stenosis in the target lesion; (2) for the target lesion with postprocedure residual stenosis of 30–50%, follow-up angiography shows >20% luminal loss compared with the prior postoperative residual angiogram in the target lesion	The device successfully released and was successfully recovered	Ischemic and hemorrhage stroke or death	6	DCB:3 BMS:3	–
Raghuram	NG	The stent was achieved in all procedures without any technical complications	NG	Median 15	NG	9
Song	NG	Residual stenosis < 30%	NG	Median 43(DES) Median 60 (BMS)	DES:12 BMS:12	9
Nicolas	>70%	Residual stenosis ≤ 30%	NG	Median 5	at least 4 weeks or 6 months	7
Feng	≥50%	The stents were all successfully released to their target positions	Ischemic and hemorrhage stroke or death	Mean 3 (DES) Mena 6 (BMS)	DES:6 weeks BMS:6 weeks	8

Author	Year	Region	Study	Inclusion criteria		Sample, *n* DCB/DES BMS	
				Stenosis area	Intervention		
Che	2018	China	Retrospective cohort	VA	DES vs. BMS	147	165
He	2019	China	RCT	VA	DES vs. BMS	20	20
Maciejewski	2019	Poland	Prospective cohort	VA	DES vs. BMS	144	270
Long Li	2020	China	Prospective cohort	VA	DES vs. BMS	76	74
Si J-H	2022	China	RCT	ICA	DES vs. BMS	92	96
Wang	2022	China	Retrospective cohort	VA	DES vs. BMS	29	12
Jia	2022	China	RCT	ICA	DES vs. BMS	132	131
Jing	2025	China	RCT	VA	DES vs. BMS	62	67

Author	Restenosis	Technical success	perioperative complications	follow-up time, months	Duration of DAPT, months	Score
Che	≥50%	Residual stenosis < 30%	NG	Mean 34.8	DES:6 BMS:at least 3	7
He	A lesion demonstrating more than 50% stenosis (within or immediately [within 5 mm] adjacent to the stent) and more than 30% absolute luminal loss at 6-month angiographic follow-up imaging (30% increase in posttreatment stenosis)	NG	NG	Median 6.45	NG	–
Maciejewski	≥50%	NG	<30%	Mean 18.9	DES:6–12 BMS:1	7
Long Li	A diameter loss 50% in the stent on vascular imaging (CT angiography or DSA) or as peak systolic velocity (PSV) 170 cm/s, end diastolic velocity (EDV) 45 cm/s, and PSV ratio 2.7	Residual stenosis < 30%	NG	Mean 12	DES:12 BMS:3	9
Si J-H	30% higher than the postopertive residual stenosis	Stent success with no stroke or death before discharge	NG	6	DES:3 BMS:3	–
Wang	≥50%	Residual stenosis < 50%	Ischemic and hemorrhage stroke and death within 30d	Mean 14.1	DES:12 BMS:3	9
Jia	≥50%	Residual stenosis < 30%	NG	12	DES:3 BMS:3	–
Jing	NG	NG	NG	6	DES:3 BMS:3	–

RCT, randomized controlled trial; ICA, intracranial artery; VA, vertebral artery; NG, no given; DCB, drug-coated balloon; DES, drug-eluting stent; BMS, bare metal stent; DAPT, dual anti-platelet therapy.

### 3.3 Quality assessment

**Figure 3:**
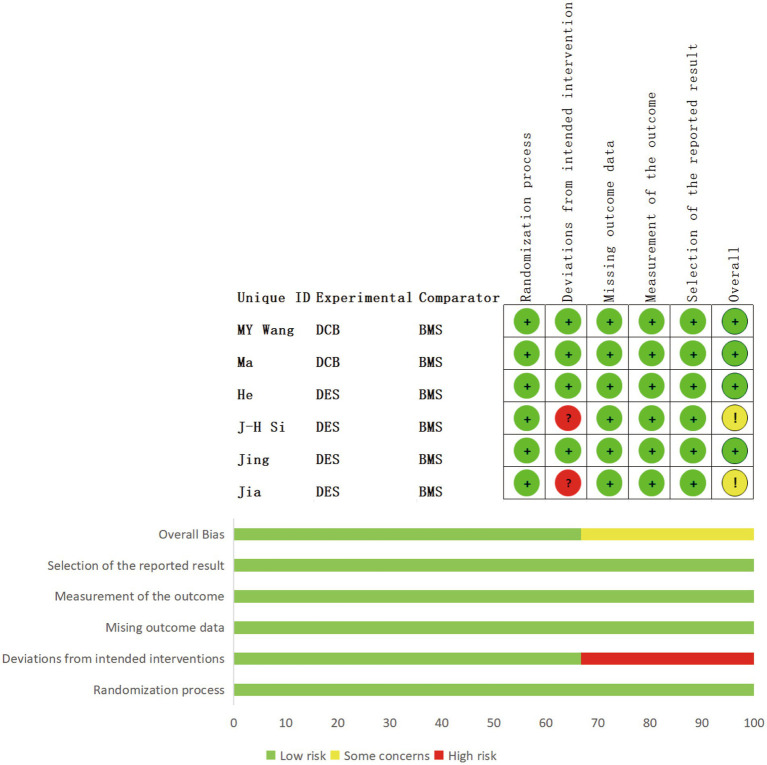
Details of quality assessment of the included RCTs.

Eleven of the included studies (27–36) were assessed using the NOS. Of these, 54.5% (6/11) scored nine points (28–31, 35, 36), 18.2% (2/11) scored eight points (27), and 27.3% (3/11) scored seven points (32–34), as shown in Table 1. Additionally, six studies (37–42) were assessed using the Rob 2.0 tool. Among these, 33.3% (2/6) showed deviations from the intended interventions, and all studies (100%, 6/6) were not blinded. The remainder were not deemed to present a significantly high risk of bias (Figure 3).

### 3.4 Result

#### 3.4.1 Restenosis

##### 3.4.1.1 Primary network meta-analysis results

Sixteen studies (26–30, 32–42) on RS were combined, involving a total of 2,305 patients and 2,361 angioplasties and stent placements (228 in the DCB group, 889 in the DES group, and 1,244 in the BMS group). The results indicated that both DCB (OR: 0.24, 95%CI: 0.10–0.57) and DES (OR: 0.37, 95%CI: 0.22–0.64) were associated with a lower risk of RS relative to BMS. However, there was no significant difference between DCB and DES regarding the reduction of RS (Table 2).

**Table 2:** League table of the primary analysis of RS.

	vs. BMS	vs. DES
DCB, OR (95% CI)	0.24 (0.10, 0.57)	0.65 (0.24, 1.77)
DES, OR (95% CI)	0.37 (0.22, 0.64)	_

##### 3.4.1.2 Heterogeneity in RS definitions

Among these trials, 12 studies (26–29, 33–39, 41) defined RS as ≥ 50%, one study (40) defined as ≥ 30%, two study (32) defined as ≥ 70%, and two studies (30, 42) provided RS rates without specifying the definition; one study (31) lacked both definition and data. To assess the impact of definition heterogeneity, we restricted the analysis to the 12 studies using a consistent ≥50% stenosis. The results (Supplementary Table 1) indicated unchanged from the primary analysis (Table 2). The overall conclusion may be unrelated to the heterogeneity of definition.

###### 3.4.1.2.1 Heterogeneity in elective procedures

Six studies were elective procedures (28, 33, 37, 38, 40, 41), which required a specific time interval between the qualifying event (e.g., stroke or TIA) and the procedures. The subgroup result observed no significant advantage for DCB vs. DES in reducing RS (Supplementary Table 2).

##### 3.4.1.3 Exploration of heterogeneity in DES vs. BMS

**Figure 4:**
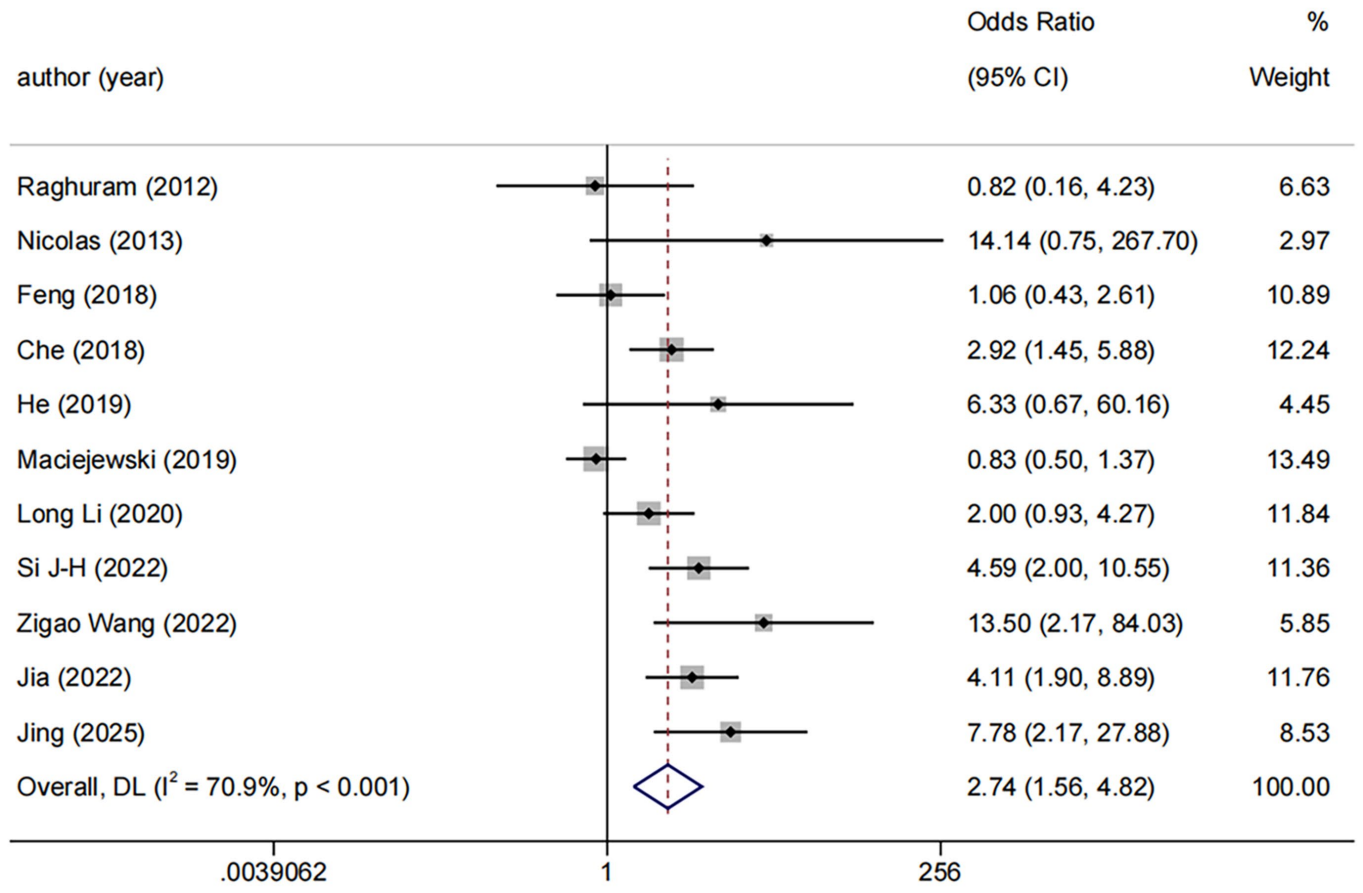
Forest plot of DES vs. BMS.

**Figure 5:**
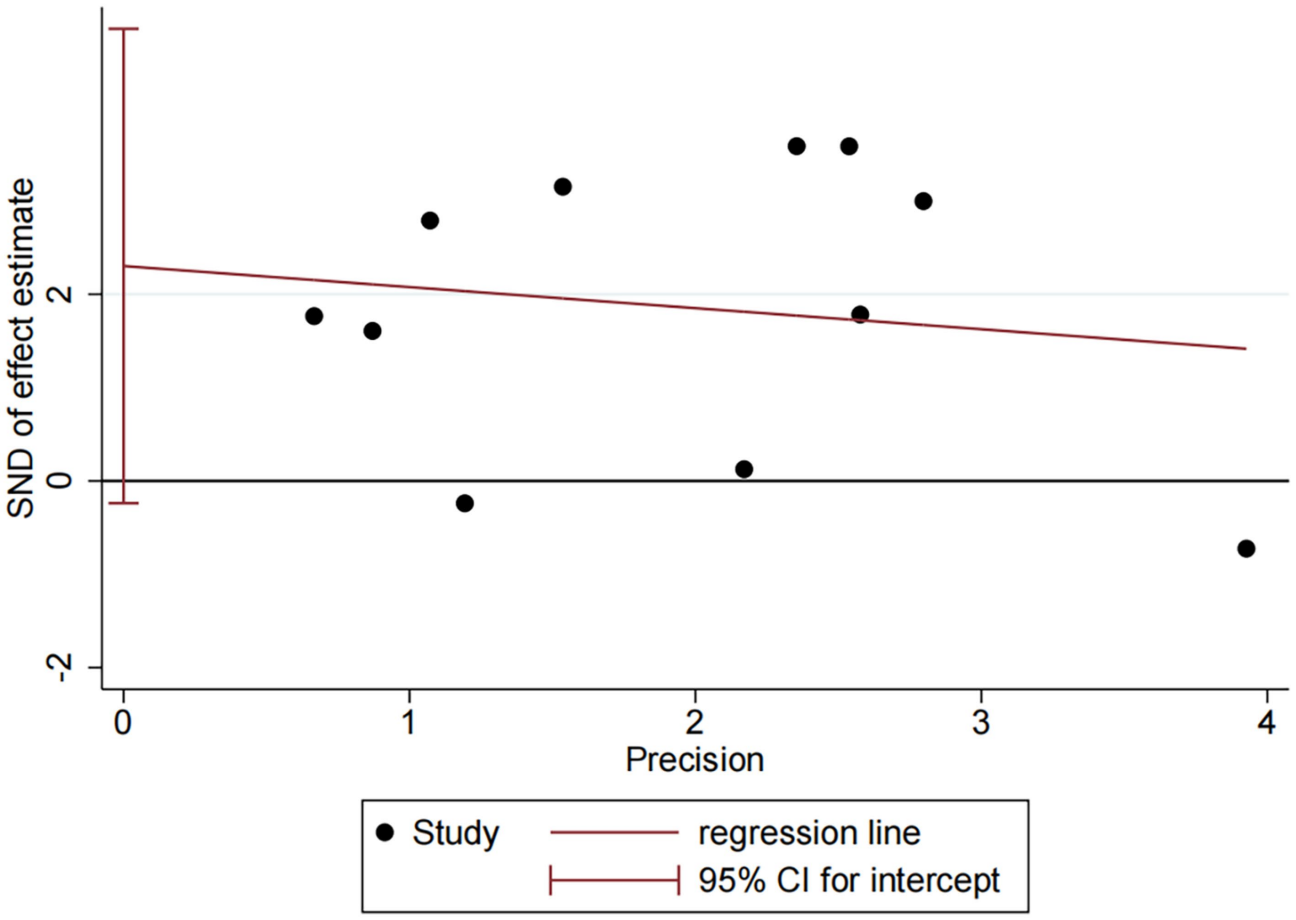
Publication bias plot of DES vs. BMS.

DES vs. BMS exhibited substantial heterogeneity (*I*^2^ = 70.9%, Figure 4). To further explore sources of heterogeneity, we performed meta-regression analyses for the comparison (*n* = 11). The analyses examined study region, lesion location, study design, and follow-up duration. None of these covariates demonstrated a statistically significant association with the treatment effect on RS (all *p* > 0.05), suggesting that the observed heterogeneity is unrelated to the covariates in our study (Supplementary Table 3). No significant publication bias was observed (Egger *p* = 0.071, Figure 5).

**Figure 6:**
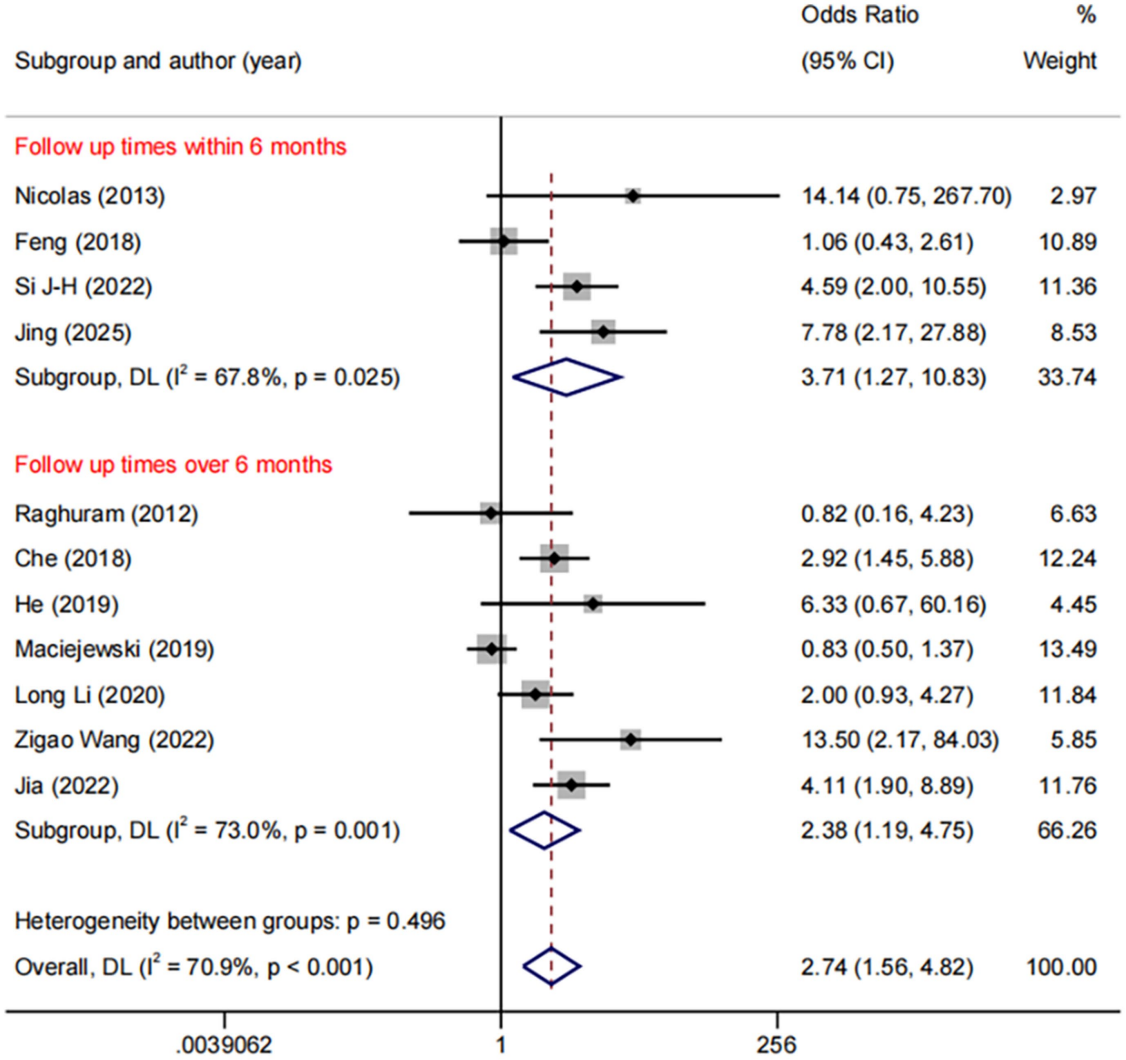
Forest plot of DES vs. BMS at different follow-up times.

Among the 11 trials comparing DES with BMS, the follow-up durations were varied: ≤6 months (*n* = 4), 6–12 months (*n* = 3), and >12 months (*n* = 4). We used ≤6 months vs. >6 months as the common cut-off for subgroup analyses. The result revealed a consistent benefit of DES over BMS in both the ≤6 months (OR: 3.71, 95%CI: 1.27–10.83, *I*^2^ = 67.8%, *p* = 0.025) and >6 months (OR: 2.38, 95%CI: 1.19–4.75, *I*^2^ = 73.0%, *p* = 0.001, Figure 6).

**Figure 7:**
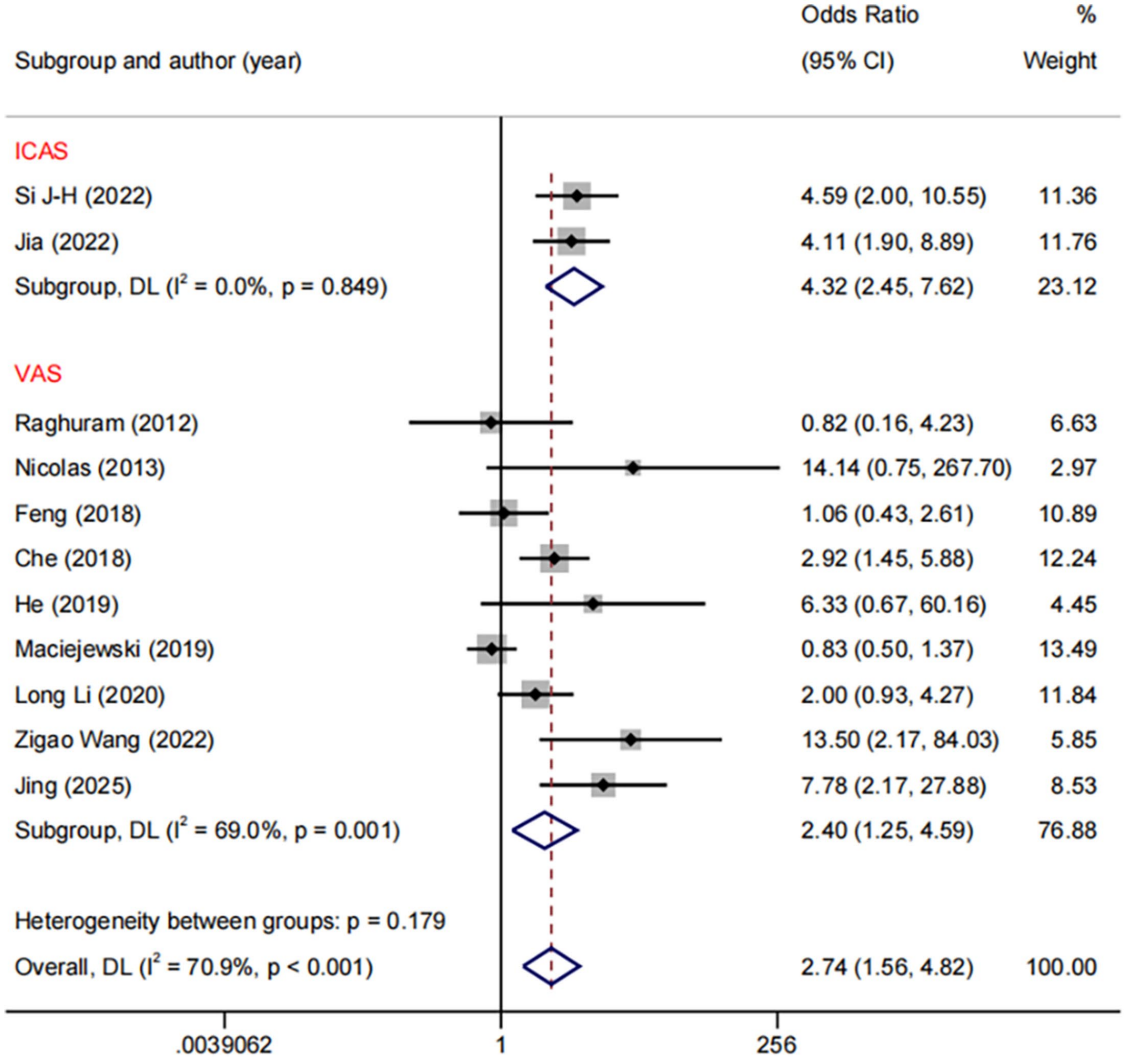
Forest plot of DES vs. BMS at different RS segments.

In the DES versus BMS comparison, we conducted subgroup analyses based on different stenotic segments. The results demonstrated a significant reduction with DES compared to BMS in restenosis of both ICA (OR: 4.32, 95%CI: 2.45–7.62, *I*^2^ = 0.0%, *p* = 0.849) and VA (OR: 2.40, 95%CI: 1.25–4.59, *I*^2^ = 69.0%, *p* = 0.001, Figure 7). Regarding the comparison between DES and BMS, our subgroup analysis across different study design revealed no heterogeneity within the RCTs subgroup (*I*^2^ = 0%, *p* = 0.006), whereas significant heterogeneity was present within the NRCS (non-randomised controlled studies) subgroup (*I*^2^ = 67.1%, *p* < 0.001) (Supplementary Figure 1).

##### 3.4.1.4 Analysis of DCB vs. BMS

**Figure 8:**
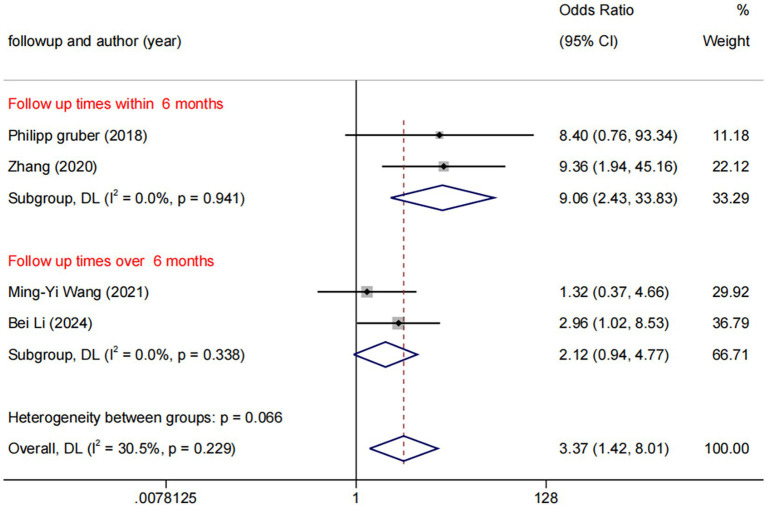
Forest plot of DCB vs. BMS at different follow-up times.

**Figure 9:**
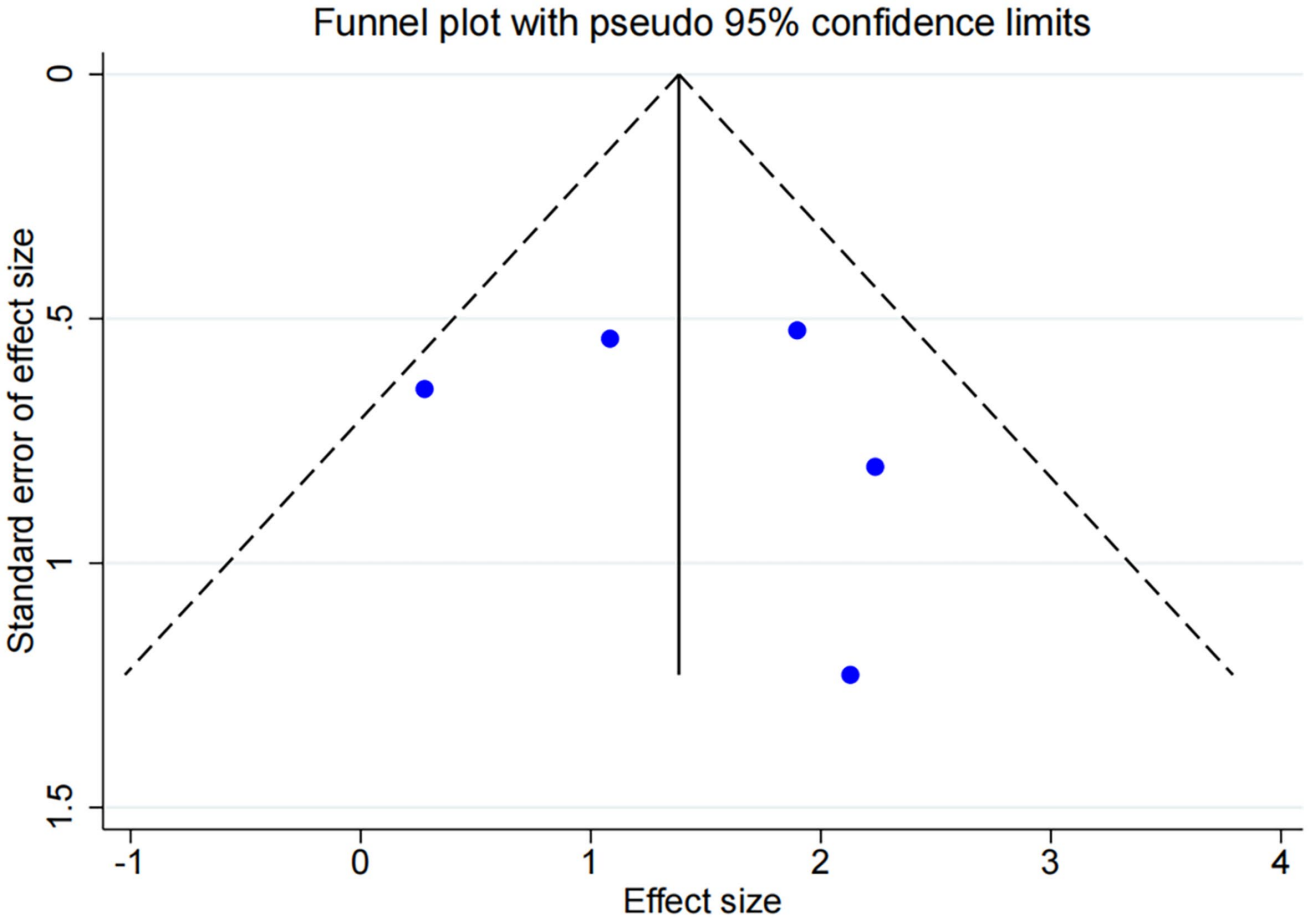
Funnel plot of DCB vs. BMS.

Among the five trials comparing DCB with BMS, the follow-up durations were fixed 6 months (*n* = 3) and 12 months (*n* = 2). Subgroup analysis revealed a statistically significant difference between DCB vs. BMS within 6 months (OR: 9.06, 95% CI: 2.43–33.83, *I*^2^ = 0.0%, *p* = 0.941), whereas no statistically significant difference was observed beyond 6 months (Figure 8). No significant publication bias was observed (Figure 9).

#### 3.4.2 Technical success and rescue procedures

Among the 17 studies included, three studies (27, 29, 36) defined residual stenosis < 50% and six studies (31–33, 35, 37, 41) defined < 30%, collectively aligning with our criterion for technical success. The results showed that there was no statistical difference among different types of instruments (Table 3).

**Table 3:** League table of technical success.

	vs. BMS	vs. DES
DCB, OR(95% CI)	0.78 (0.23, 2.73)	0.57 (0.14, 2.32)
DES, OR(95% CI)	1.37 (0.73, 2.60)	_

The 12 studies (26–29, 32–37, 41, 42) reported rescue events. Across these trials, rescue procedures occurred in 0.7–10.8% of patients per arm. The significant differences were found only in the DCB vs. BMS comparison (OR: 3.41, 95%CI: 1.13–10.25, Supplementary Table 4).

#### 3.4.3 Perioperative complications

Eleven studies were included in the analysis (26–29, 34–38, 40, 41). Results indicated that DCB was associated with lower risks compared to BMS (OR: 0.33, 95%CI: 0.12–0.93) and DES (OR: 0.43, 95%CI: 0.19–0.95) (Table 4).

**Table 4:** League table of perioperative complications.

	vs. BMS	vs. DES
DCB, OR(95% CI)	0.43 (0.19, 0.95)	0.33 (0.12, 0.93)
DES, OR(95% CI)	1.29 (0.67, 2.51)	_

## 4 Discussion

Our analysis yielded several key observations. Firstly, DCB and DES demonstrated comparable efficacy in reducing restenosis rates, both outperforming BMS. DCB exhibited stronger short-term efficacy that may diminish subsequently, whereas DES may maintain more consistent long-term benefits. Second, DCB was associated with lower perioperative complication rates but necessitated greater reliance on rescue stenting. These findings support a cautiously optimistic and individualized approach to the use of DCB and DES in clinical practice.

### 4.1 Transient efficacy and underlying mechanisms of DCB

The DCB may provide a transient reduction in RS compared to the sustained benefit of DES. The drug typically coated on DCB is paclitaxel, which acts by stabilizing microtubules, enhancing microtubule polymerisation, and inhibiting their depolymerization, thereby arresting cells in the M-phase of the mitotic cycle (43). The release pattern of paclitaxel is characterized by an initial “burst release” followed by a plateau phase. This explains the significant reduction in RS observed during early follow-up times, a benefit consistently demonstrated across multiple studies (38, 44). Our subgroup analysis suggested that BMS may be associated with a higher risk of RS compared to DCB within 6 months (OR: 9.06). However, DCB may provide transient drug exposure and balloon-based mechanical expansion, but cannot provide the long-term mechanical support capability over 6 months. Consequently, once the pharmacological effect diminishes, the vessel may undergo delayed neointimal hyperplasia (“catch-up” phenomenon) or constrictive remodeling (45), and the DCB’s advantage in reducing RS may consistently diminish after 6 months’ follow up. As the long-term follow-up study indicated, DCB alone may be insufficient for sustaining satisfactory long-term patency (36).

### 4.2 The dilemma of residual stenosis and rescue procedures

The “implant-free” strategy in DCB angioplasty presents a set of unique intraoperative challenges. Notably, the balloon angioplasty typically achieves only moderate vassal expansion, reflecting the clinical preference to avoid arterial dissection. Our analysis corroborated this: in 40% of DCB studies, technical success was defined as residual stenosis < 50%, whereas 41.7% of DES studies employed the stricter < 30% criterion. Research (46) indicated that aggressive angioplasty correlated with significantly higher dissection rates (3.6% vs. 17.7%, *p* = 0.018) and bailout stenting rates (7.3% vs. 21.0%, *p* = 0.040). We similarly indicated that DCB was more strongly associated with rescue procedures (OR: 3.41) compared with BMS. These findings were corroborated by a randomized controlled trial (6), which demonstrated that aggressive balloon angioplasty (balloon diameter 50–70% of the proximal artery diameter) combined with intensive medical therapy yielded better outcomes than intensive medical therapy alone. Nevertheless, the incidence of arterial dissection in that trial was consistent with a previous meta-analysis of submaximal angioplasty for intracranial atherosclerotic disease (47).

### 4.3 Perioperative safety and simplified pharmacotherapy

Despite the limitations such as transient efficacy and the risk of requiring rescue procedures, the DCB strategy offered significant compensatory advantages. Our analysis indicated that DCB was associated with a lower risk of perioperative complication compared to both DES (OR: 0.33, 95%CI: 0.12, 0.93) and BMS (OR: 0.43, 95%CI: 0.19, 0.95), which highlights its better short-term safety profile. This safety advantage, coupled with the absence of a permanent implant, reduced the postoperative duration of dual antiplatelet therapy (DAPT). This characteristic was reflected in our analysis: 60% of the included studies of DCB vs. BMS reported DAPT durations of merely 3 months. The STOPDAPT-2 trial (48) supported this approach, indicating that 1-month DAPT after DCB angioplasty significantly reduced bleeding risk without increasing cardiovascular events compared to 12-months DAPT. Accordingly, DCB is an excellent choice for patients at high risk of bleeding.

### 4.4 Sustained efficacy and long-term considerations of DES

DES consists of a metallic or synthetic scaffold coated with a polymer matrix that carries and gradually releases anti-proliferative drugs such as sirolimus, which acts primarily by inhibiting the mTOR signaling pathway, thereby preventing vascular smooth muscle cell proliferation and migration and arresting the cell cycle at the G1-to-S phase transition (49). The combined effect of mechanical scaffolding and sustained drug release allows DES to maintain low RS rates. Consistent with this mechanism, our analysis indicated this, demonstrating a consistent benefit of DES over BMS in both the ≤ 6 months and > 6 months subgroups, as well as in both ICA and VA locations. Moreover, DES faced unique challenges in specific anatomical locations. Our subgroup analysis of VAS demonstrated significant therapeutic efficacy (OR: 2.40), exhibited marked heterogeneity (*I*^2^ = 69.0%). This heterogeneity may be partially explained by anatomical factors. The peculiar anatomy of the vertebral artery both facilitates in-stent atherosclerotic plaque accumulation (50, 51) and induces passive stretching due to respiratory motion (52), collectively increasing the risk of late stent fractures (53). When applying DES in VAS lesions, clinicians must conduct individualised assessments based on the patient’s specific anatomical characteristics to balance efficacy and safety. In addition, in resource-constrained settings (such as where DES is unavailable), DCB therapy may be prioritised. Nevertheless, we look forward to future trials exploring the comparative health economics of these three interventions.

### 4.5 Future directions

We hope that future studies will further explore the influence of stenosis or implantation site characteristics. Specifically, bifurcation and arterial curvature, as well as the distinction between single and overlapping stenting. The interpretation of our findings must account for the predominance of China populations in the included literature, suggesting caution is warranted when translating these findings across geographical regions. Furthermore, giving the differences in clinical management and device specifications across healthcare systems, the observed efficacy requires further validation in diverse populations.

### 4.6 Limitations of the study

Due to the star network geometry employed in our analysis, the 16 studies included in the RS analysis did not employ a uniform definition of this outcome. Although our *post hoc* analysis confirmed the stability of the primary results, the potential for bias introduced by these varying definitions cannot be fully ruled out. Second, although we conducted subgroup analyses based on follow-up duration, and these analyses did not indicate that follow-up duration influenced the primary outcome of DCB vs. BMS, we nevertheless anticipate studies exploring the efficacy of DCB beyond 1 year. Additionally, the majority of studies included in the meta-analysis originated from China, which may introduce potential limitations. Our analysis on vertebral artery stenosis was limited by insufficient data in the DCB group. The inclusion of both RCTs and NRCTs further posed a challenge to the overall quality and consistency of this study.

## 5 Conclusion

In this network meta-analysis, both DCB and DES were associated with reducing RS rates compared with BMS. Regarding safety, DCB demonstrated a potential advantage over both BMS and DES in reducing perioperative complications. However, DCB was associated with a higher likelihood of patients requiring rescue surgery compared with BMS. Subgroup analyses revealed that at the 6-month follow-up, both DCB and DES demonstrated significant efficacy compared with BMS. The advantage of DCB failed to persist, whereas the benefit of DES extended over a longer timeframe. DCB and DES possess their own distinct advantages. Further RCTs are required to validate our findings.
