# Supplementary figures and images for: Net meta-analysis: comparison of bare metal stent, drug-coated balloon and drug-eluting stent in the treatment of cerebral arterial stenosis
Source: Front Neurol. 2026 Jan 5;16:1637301. doi: 10.3389/fneur.2025.1637301 (PMC12812648; doi:10.3389/fneur.2025.1637301)

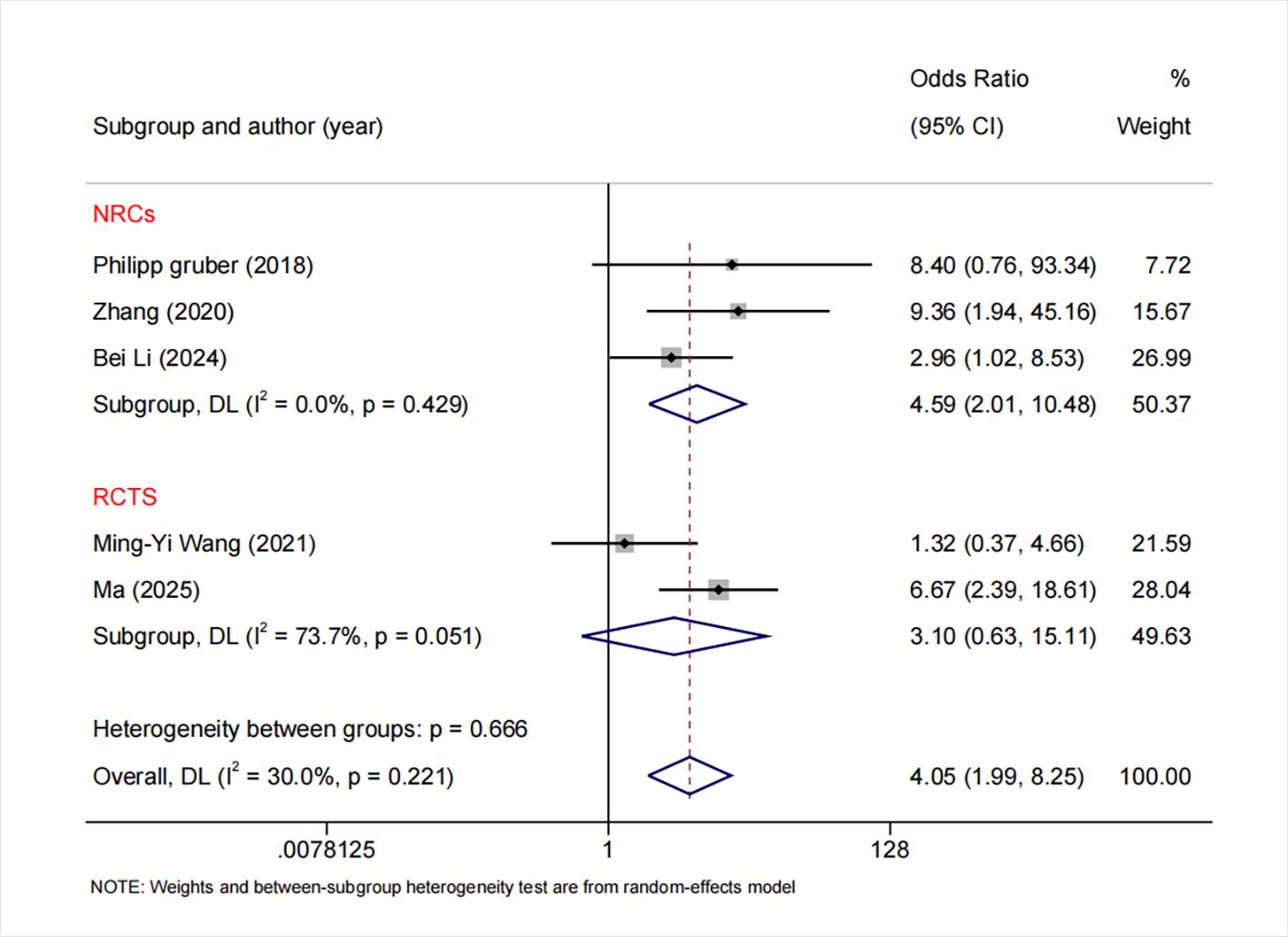

Supplement: Supplementary file 1 [file Image_1.TIF]
