# Supplementary material for: Net meta-analysis: comparison of bare metal stent, drug-coated balloon and drug-eluting stent in the treatment of cerebral arterial stenosis
Source: Front Neurol. 2026 Jan 5;16:1637301. doi: 10.3389/fneur.2025.1637301 (PMC12812648; doi:10.3389/fneur.2025.1637301)
Supplement: Supplementary file 2 [file Supplementary_material.docx]

**Supplementary Table 1.** League table of post-hoc analysis

|  | **vs. BMS** | **vs. DES** |
| --- | --- | --- |
| **DCB, OR(95% CI)** | 0.24 (0.11, 0.56) | 0.55 (0.20, 1.53) |
| **DES, OR(95% CI)** | 0.44 (0.24, 0.83) | **_** |

**Supplementary Table 2.** League table of Elective procedures

|  | **vs. BMS** | **vs. DES** |
| --- | --- | --- |
| **DCB, OR(95% CI)** | 0.24 (0.10, 0.56) | 1.00 (0.32, 3.14) |
| **DES, OR(95% CI)** | 0.24 (0.11, 0.49) | **_** |

**Supplementary Table 3.** Meta-regression results for RS

| **Variable** | **SE** | **t** | **P value** |
| --- | --- | --- | --- |
| **Region** | 0.54 | -2.17 | 0.058 |
| **Location** | 0.67 | -0.93 | 0.377 |
| **Design** | 0.48 | -2.23 | 0.053 |
| **Followup** | 0.62 | -0.67 | 0.517 |

**SE: Standard Error**

**Supplementary Table 4.** League table of Rescue procedures

|  | **vs. BMS** | **vs. DES** |
| --- | --- | --- |
| **DCB, OR(95% CI)** | 3.41 (1.13, 10.25) | 3.26 (0.64, 16.62) |
| **DES, OR(95% CI)** | 1.04 (0.32, 3.46) | **_** |
